# Pesticides Decrease Bacterial Diversity and Abundance of Irrigated Rice Fields

**DOI:** 10.3390/microorganisms8030318

**Published:** 2020-02-25

**Authors:** Michael Onwona-Kwakye, Kimberly Plants-Paris, Kadiatou Keita, Jessica Lee, Paul J. Van den Brink, Jonathan N. Hogarh, Charles Darkoh

**Affiliations:** 1 Aquatic Ecology and Water Quality Management Group, Wageningen University, P.O. Box 47, 6700 AA Wageningen, The Netherlands; mokatyk@gmail.com (M.O.-K.); Paul.vandenBrink@wur.nl (P.J.V.d.B.); 2 Ghana Environmental Protection Agency, P.O. Box, Accra M326, Ghana; 3 Center for Infectious Diseases, Human Genetics, and Environmental Sciences, Department of Epidemiology, School of Public Health, University of Texas Health Science Center, Houston, TX 77030, USA; Kimberly.S.PlantsParis@uth.tmc.edu (K.P.-P.); Kadiatou.Keita@uth.tmc.edu (K.K.); Jessica.N.Lee@uth.tmc.edu (J.L.); 4 Wageningen Environmental Research, P.O. Box 47, 6700 AA Wageningen, The Netherlands; 5 Department of Environmental Science, College of Science, Kwame Nkrumah University of Science and Technology, Kumasi PMB University Post Office, Kumasi 400800, Ghana; jhogarh@gmail.com; 6 MD Anderson Cancer Center UTHealth Graduate School of Biomedical Sciences, Microbiology and Infectious Diseases Program, Houston, TX 77030, USA

**Keywords:** pesticides, soil microbiota and diversity, irrigated fields, soil bacteria, pesticides and bacteria

## Abstract

Bacteria play an important role in soil ecosystems and their activities are crucial in nutrient composition and recycling. Pesticides are extensively used in agriculture to control pests and improve yield. However, increased use of pesticides on agricultural lands results in soil contamination, which could have adverse effect on its bacterial communities. Here, we investigated the effect of pesticides commonly used on irrigated rice fields on bacterial abundance and diversity. Irrigated soil samples collected from unexposed, pesticide-exposed, and residual exposure areas were cultured under aerobic and anaerobic conditions. DNA was extracted and analysed by 16S rRNA sequencing. The results showed overall decrease in bacterial abundance and diversity in areas exposed to pesticides. Operational taxonomic units of the genera *Enterobacter*, *Aeromonas*, *Comamonas*, *Stenotrophomonas*, *Bordetella*, and *Staphylococcus* decreased in areas exposed to pesticides. Conversely, *Domibacillus*, *Acinetobacter*, *Pseudomonas*, and *Bacillus* increased in abundance in pesticide-exposed areas. Simpson and Shannon diversity indices and canonical correspondence analysis demonstrated a decrease in bacterial diversity and composition in areas exposed to pesticides. These results suggest bacteria genera unaffected by pesticides that could be further evaluated to identify species for bioremediation. Moreover, there is a need for alternative ways of improving agricultural productivity and to educate farmers to adopt innovative integrated pest management strategies to reduce deleterious impacts of pesticides on soil ecosystems.

## 1. Introduction

Microbes play an important role in soil ecosystems and their activities are critical in nutrient composition and recycling [1,2,3]. The increasing global human population (expected to be approximately 9.7 billion by 2050) would dramatically increase the demand for food resources [4]. The increase in demand for food throughout the world has prompted farmers to devise ways to increase productivity, including the use of pesticides. Increased use of pesticides on agricultural lands causes contamination of the soil ecosystem with toxic chemicals [5]. Modern agriculture largely relies on extensive application of agrochemicals such as inorganic fertilizers and pesticides. Indiscriminate long-term pesticides use or over-application of pesticides could have severe effects on soil ecosystems, which may lead to alteration and/or erosion of beneficial soil microflora [6]. Annually, an estimated two million tons of pesticides are applied on agricultural lands worldwide [7]. In 2012, herbicides accounted for 49% of chemicals used in agriculture and this was followed by fumigants (19%), insecticides (18%), and fungicides (14%) [8].

Pesticides may also affect non-target organisms and exert deleterious effects on the environment and farmland biodiversity [9,10]. Among the non-target populations, soil microorganisms are extremely important, since they play an essential role in nutrient turnover [11] and maintain generative capacities of agroecosystems [12]. The impact of pesticides on soil bacterial populations could also be used as potential indicators of their toxicity and alteration of the environment [13]. Metabolites or the degraded products of pesticides can persist in the soil long-term. For example, trifloxystrobin typically has a half-life of 7 days in the soil, whereas its metabolite (E,E)-trifloxystrobin acid) has a half-life of up to 268 days [13]. Previous studies on tebuconazole and carbendazim indicated that increase in concentration of these pesticides can affect soil microbial activity. Specifically, increasing concentrations of moderate to high doses of tebuconazole significantly inhibited soil respiration and enzymatic activities [13]. Further, moderate doses of carbendazim stimulated urease and invertase activities and significantly inhibited other soil bacterial activities after 7 days [13].

Rice is a major source of food for more than half of the world’s population [14]. However, rice cultivation is usually vulnerable to a variety of pests and requires pesticides to help control them and improve yield. Although, pesticides help increase economic gains from agriculture, they also impact bacterial ecosystems in the soil. Due to the large amount of pesticides applied during rice cultivation, the rice field ecosystem is one of the major contributing agroecosystems from which pesticide residues contaminate the environment [15]. Although, pesticides are commonly used to improve agricultural yields, little is known about their effects on the soil microbiota in irrigated rice fields. The goal of this study was to investigate the effect of pesticides commonly used on irrigated rice fields on bacterial abundance and diversity. Here, an irrigated rice field in Ghana was used as a case study. This is because majority of the rice farms in Ghana are irrigated and pesticides are often applied on these irrigated fields.

## 2. Materials and Methods

### 2.1. Study Area

The samples used in the study were collected from Kpong irrigation project site at Akuse, Ghana, where rice is cultivated all year round under well-managed irrigation schemes. Sample collection was limited to a 4-hectare irrigated rice field with known history of pesticides use during the growing season (Table 1). The climate is the savannah type, characterized by bimodal rainfall pattern ranging from 900 to 1100 mm annually with a predominant wind speed between 1 and 2 knots. The mean annual temperature is 28.6 °C. The soil in this area is heavy dark clay with high water holding capacity of up to 220 mm per meter depth of soil and an average dry bulk density of about 1.0 g/cm^3^ [16]. Water is sourced from a lake upstream through canals and via laterals to cover the fields. Jasmine 85 is the variety of rice cultivated and takes 110–120 days to mature, usually starting from June/July-October/November. The samples analysed in this study were collected in November 2016.

### 2.2. Sampling Procedure for Soil

Wet soil samples (5–10 g) were collected from different locations along the irrigation canal from the water source upstream, the rice field itself (where the chemicals are applied), and areas downstream of the irrigation canal. The soil samples were collected using soil auger (5.1 cm in diameter and 122 cm in length) at depths between 15 and 30 cm and grouped as (i) water source upstream (unexposed); (ii) rice field where the chemicals were applied (exposed); (iii) areas downstream of the irrigation line (residual). Eleven [11] samples were collected at equal intervals of 25 m at each depth: unexposed (samples 1–4; from the water source upstream), exposed (samples 5–9; rice field where the pesticides are applied), and residual zone (samples 10 and 11; areas downstream of the irrigation line).

The soil samples were refrigerated and shipped on ice-packs for analysis at University of Texas Health Science Center, School of Public Health, Department of Epidemiology, Human Genetics, and Environmental Sciences, Center for Infectious Diseases, Houston, TX, USA.

### 2.3. Bacteria Culture

Anaerobic condition was maintained in a Bactron 600 anaerobic chamber (Sheldon Manufacturing, Cornelius, OR) using 5% CO_2_, 10% H_2_, and 85% N_2_. The soil samples (1 g each) was suspended in 20 mL of brain heart infusion (BHI) medium (Becton Dickinson, Franklin Lakes, NJ). To isolate both aerobic and anaerobic bacteria in the soil, the suspensions were divided into two in 50 mL culture tubes and one tube (10 mL) was incubated aerobically or anaerobically, respectively, at 37 °C for 24 h. Following the 24-h incubation period, the culture was thoroughly mixed and freezer stocks (1 mL) of each culture were made in 10% DMSO and stored at −80 °C. The remaining culture was centrifuged for 10 min at 15,000× *g* and the pellets were stored at −20 °C for DNA isolation and PCR analysis.

### 2.4. DNA Extraction and 16S Ribosomal RNA (rRNA) Gene Sequencing

DNA was isolated from each of the bacterial pellets using the GenElute Bacterial Genomic DNA Kit (Sigma-Aldrich, St. Louis, MO, USA), according to the protocol provided by the manufacturer. The concentration and purity of the extracted DNA was determined using NanoDrop (ThermoScientific, Wilmington, DE, USA) and the DNA quality was assessed by agarose gel electrophoresis. The extracted DNA samples were normalized and equal amounts were analysed by 16S ribosomal RNA (rRNA) gene sequencing. The V4 region of the bacterial 16S rRNA gene was PCR-amplified using bacteria/archaeal primers 515F (5′GTGCCAGCMGCCGCGGTAA3′) and 806R (5′GGACTACHVGGGTWTCTAAT3′) [17]. The conditions for amplification were: 1 cycle of 94 °C for 3 min, 35 cycles of 94 °C for 45 s, 50 °C for 60 s, and 72 °C for 90 s, and 72 °C for 10 min [17]. Sequencing was performed at the Alkek Center for Metagenomics and Microbiome Research (Baylor College of Medicine, Houston, Texas) on the Illumina MiSeq platform (Illumina, San Diego, CA, USA) using 2 × 250 bp paired-end protocol, which yielded pair-end reads that almost completely overlapped, targeting at least 15,000 reads per sample. DNA extracted under similar conditions, but without any bacterial pellet was used as control. The read pairs were demultiplexed based on unique molecular barcodes, and merged using USEARCH v7.0.1001 [18]. The data was analysed using the CMMR-16S (v4) analytic pipeline, as described previously [17,19,20]. The CMMR pipeline for 16S analysis leverages the QIIME (Quantitative Insights Into Microbial Ecology) software package [17,20] and custom analytic packages. The 16S rRNA gene sequences were clustered into taxonomic operation units (OTUs) at a similarity cut-off value of 97% using the UPARSE algorithm in QIIME and the SILVA database [21]. The OTUs were determined by mapping to the SILVA database containing only the 16S V4 region to determine taxonomies [21]. An OTU table was constructed for taxonomic summaries and the alpha- and beta-diversity calculated [22]. The data from this study will be deposited in the U.S. National Center for Biotechnology Information and will be available through accession number PRJNA608009.

### 2.5. Data Analysis

Data were analysed using STATA 15 for Windows (StataCorp LLC, College Station, TX) and R software [21]. To visualize the frequency of genera across soil samples, heat maps derived from the relative abundance of the OTUs were generated using the Heatplus, gplots, and RcolorBrewer packages for R [21]. To assess the association between region of pesticide exposure and frequency of selected genera, the Kruskal-Wallis test was used. Statistical significance was defined as *p*-value < 0.05.

The bacterial data was also analysed using multivariate ordination techniques to assess the effects of depth, culture under either aerobic or anaerobic conditions, and exposure on the composition of the bacterial community. Genus level data were log (x + 1) transformed to down-weight the high abundances and approximate a normal distribution of the data. Since the data were compositional (relative), canonical correspondence analysis (CCA) was used [23,24]. First, a CCA using sites as explanatory variables and depth and being aerobic or anaerobic were included as covariable in order to get an overview on the (dis) similarity in genera composition between the sites. This analysis was followed by a Monte Carlo permutation test, permuting the samples within the blocks defined by covariables. Three more Monte Carlo permutation tests were performed to test the significance of depth, being cultured under aerobic or anaerobic conditions and exposure. In each test, one factor was included as explanatory variable and the two others as covariable, which defined the blocks within which the samples were permuted. A second CCA analysis was performed using the interaction between exposure and culture under aerobic or anaerobic conditions as explanatory variables and depth as covariable, in order to show the (interactive) effects of the variables. All analysis were performed using the CANOCO Software package, version 5 [24].

## 3. Results

The active ingredients, formulations, mode and frequency of application, and seasonal application rates of the pesticides used on the irrigation fields are shown in Table 1. These pesticides are applied 3 to 5 times during the season and the formulations include herbicides, insecticides, and fungicides.

### 3.1. Effect of Pesticides on Soil Bacterial Abundance and Diversity

**Figure 1:**
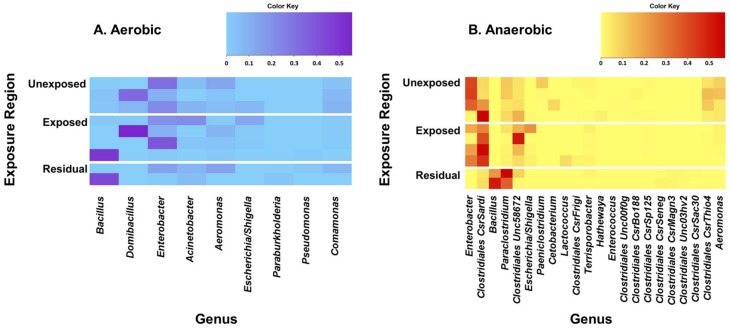
Heat maps of the predominant bacterial genera detected from the soil samples incubated under aerobic (**A**) and anaerobic (**B**) conditions. Pesticides-treated irrigated soil samples were collected from the unexposed, pesticide-exposed, and residual exposure areas and incubated for 24 h under aerobic and anaerobic conditions and analyzed for bacterial diversity. DNA was extracted and analyzed by 16S rRNA sequencing. Genera were excluded from the heat map if the greatest relative frequency among the samples was less than 0.5%. Obligate anaerobic genera were excluded from the aerobic heat map, and obligate aerobic genera were excluded from the anaerobic heat map. Genera were sorted by greatest sample-wide frequency to lowest sample-wide frequency.

The five most prevalent bacterial genera identified from the soil samples that were incubated under aerobic conditions were *Bacillus*, *Domibacillus*, *Enterobacter*, *Acinetobacter*, and *Aeromonas* (Figure 1). *Domibacillus*, *Enterobacter*, and *Aeromonas* were the most predominant genera detected from the areas that were not exposed to pesticides. On the other hand, *Bacillus*, *Domibacillus*, and *Enterobacter* were the most frequent genera identified from the samples collected from the pesticide-exposed areas whereas *Bacillus* was the most frequent in the residual exposure areas. All of the five most prevalent aerobic bacterial genera identified contain species that are reported to play beneficial roles in the soil (Table 2). The five most prevalent genera detected in the samples cultured under anaerobic conditions were *Enterobacter*, *Clostridiales* CsrSardi, *Bacillus*, *Paraclostridium*, and *Clostridiales* Unc58672. *Enterobacter*, *Clostridiales* CsrSardi, and *Paraclostridium* were the most frequent genera in the unexposed area. Clostridiales CsrSardi, *Clostridiales* Unc58672, and *Enterobacter* were the most frequent genera in the area exposed to pesticides whilst *Paraclostridium* and *Bacillus* were the most predominant genera in the residual exposure area (Figure 1).

**Figure 2:**
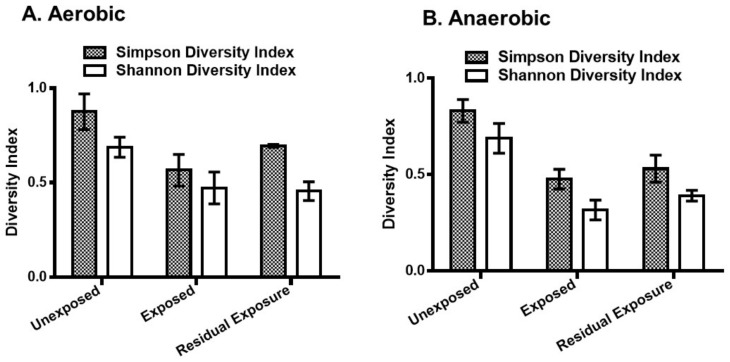
The Simpson and Shannon bacterial diversity indices of pesticide-treated irrigated soil samples. Pesticides-treated irrigated soil samples were collected from the unexposed, pesticide-exposed, and residual exposure areas and incubated for 24 h under aerobic (**A**) and anaerobic (**B**) conditions. DNA was extracted and analyzed by 16S rRNA sequencing. In the anaerobic samples, two-sample t-test showed that the mean Simpson and Shannon diversity indices were significantly different between the pesticide-exposed and unexposed areas (*p* = 0.00003 and *p* = 0.00005, respectively). In the aerobic samples, the mean Simpson and Shannon diversity indices were also significantly different between the pesticide-exposed and unexposed areas (*p* = 0.001 and *p* = 0.003, respectively). The error bars represent the mean ± S.D. of the indices of the replicate samples from each exposure group.

Both Simpson and Shannon diversity indices indicated a decrease in bacterial diversity in the pesticide-exposed area (Figure 2). Simpson diversity index showed a significant decrease in bacterial diversity in the exposed [*p* = 0.001 (aerobic), *p* = 0.00003 (anaerobic)] and the residual exposed areas [*p* = 0.022 (aerobic), *p* = 0.015 (anaerobic)]. The Shannon diversity index also showed a similar degree of significant decrease in the areas exposed to pesticides compared to the unexposed areas.

**Figure 3:**
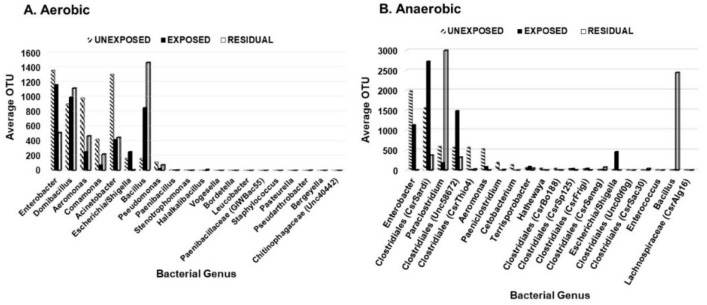
Distribution of the 20 most prevalent genera based on the region of pesticide exposure. (**A**) aerobic bacteria; (**B**) anaerobic bacteria.

To investigate the effect of the pesticides on bacterial abundance, twenty most frequent aerobic and anaerobic bacterial genera were examined based on their average operational taxonomic units (OTU). *Enterobacter*, *Aeromonas*, *Comamonas*, *Stenotrophomonas*, *Bordetella*, and *Staphylococcus* decreased in the area exposed to pesticides. The abundance of *Aeromonas* species decreased in the area exposed to pesticides but showed a slight increase in the residual exposure area (Figure 3). *Escherichia/Shigella* had the greatest frequency in areas exposed directly to pesticides. The frequency of *Bacillus* was higher in the residual area than in areas that were either exposed or unexposed to pesticides. Other anaerobic genera that significantly decreased in abundance in the area exposed to pesticides but to a lesser extent than *Enterobacter*, *Aeromonas*, *Comamonas*, *Stenotrophomonas*, *Bordetella*, and *Staphylococcus* included *Clostridiales* (CsrThio4), *Paeniclostridium*, *Clostridiales* (CsrSardi), *Paraclostridium*, *Clostridiales* (Unc58672), *Terrisporobacter*, *Clostridiales* (CsrSp125), *Clostridiales* (CsrFrigi), *Clostridiales* (CsrSeneg), and *Clostridiales* (CsrSac30). For the aerobic bacteria, the genera whose abundance decreased in the pesticides-exposed area were *Enterobacter* and *Comamonas*. Both genera are ubiquitous and contain bacterial species that play beneficial roles in the soil. On the other hand, *Domibacillus*, *Pseudomonas*, and *Bacillus* were in higher abundance in the pesticide-exposed area but to a lesser extent than *Aeromonas* in the unexposed area.

### 3.2. Multivariate Analyses

**Figure 4:**
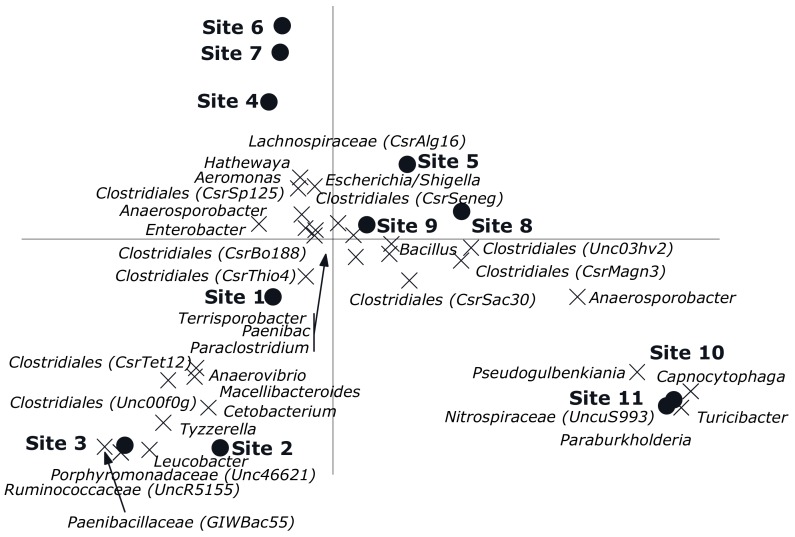
Canonical correspondence analysis biplot showing the results of the analysis using sites as explanatory variables and depth and being aerobic or anaerobic as covariables. Of all variance, 38% was explained by the differences between sites while the covariables explained 15% of the variation in genus composition. Of the variation explained by sites, 33% is displayed on the horizontal axis and an additional 18% on the vertical one. Only the 33 genera of which more than 15% of its variation is displayed by the axes are shown. Sites 1–4 (Unexposed area, sites from the water source upstream); Sites 5–9 (Exposed area, rice field where the pesticides are applied); Sites 10–11 (Residual area, sites downstream of the irrigation line).

**Figure 5:**
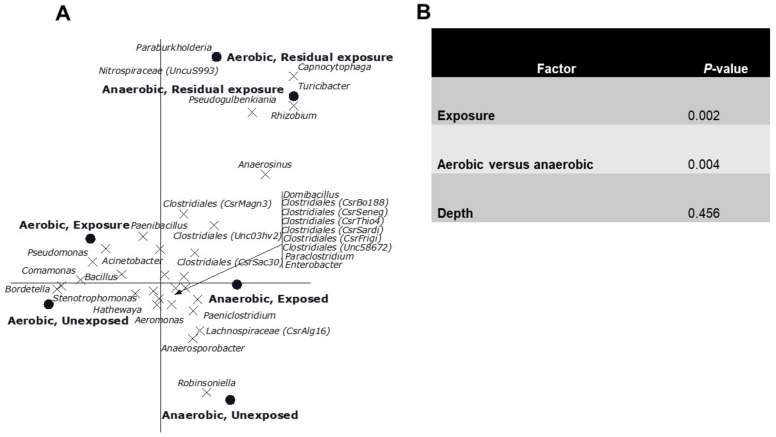
(**A**). Canonical correspondence analysis biplot showing the results of the analysis using the interaction between exposure and being aerobic or anaerobic as explanatory variables and depth as covariable. Of all variance, 32% was explained by the differences between sites while the covariables explained 2% of the variation in genus composition. Of the variation explained by the explanatory variables, 41% is displayed on the horizontal axis and an additional 32% on the vertical one. Only the 32 genera of which more than 15% of its variation is displayed by the axes are shown. (**B**). Significance of the effects of the different factors on the genus composition of the bacterial community as assessed by Monte Carlo permutation tests.

The CCA showed a clear gradient (sites, *p* = 0.002) from site 1 till 11 with different genera composition of the bacterial community was observed (Figure 4). Sites 4, 6 and 7 had relatively low numbers, which also happens to be the exposed region. Of all variance, 38% was explained by the differences between sites, while the covariables explained 15% of the variation in genus composition. A reverse CCA using depth and being cultured under ae/anaerobic conditions as explanatory variables and site as covariable resulted in a biplot showing a clear separation between depths and being cultured under ae/anaerobic conditions (*p* = 0.002). Of all variance 32% was explained by the differences between sites, while the covariables explained 2% of the variation in genus composition (Figure 5A). The results of the Monte Carlo permutation tests (Figure 5B) demonstrated that exposure and either aerobic or anaerobic culture has significant effect on the bacterial community composition. These results also indicate that the bacterial community does not recover in the residual section, with a more different composition than that of the areas directly exposed to pesticides.

## 4. Discussion

In this study, we investigated the effect of pesticides commonly used in irrigated rice fields on bacterial abundance and diversity. The results showed that the use of pesticides decreased the abundance and bacterial diversity of the soil. Soil samples collected from three locations (unexposed, exposed, residually-exposed areas) within an irrigated rice field with a history of pesticide use were examined for the presence of aerobic and anaerobic bacteria. The data demonstrated that aerobic bacteria exhibited a higher return to diversity in the residual pesticide exposure areas, compared to the anaerobic residual exposure areas (Figure 2).

Among the top 20 most frequently identified aerobic genera (Figure 3A), fourteen contain species that are known to be beneficial to the soil (Table 2), seventeen genera contain species that are potential pathogens, whereas fifteen contain species that are both beneficial to the soil and pathogenic (Table 2). Three of the genera (*Domibacillus*, *Halalkalibacillus*, *Vogesella*) contain novel soil bacteria with no established roles in the soil [28,45,46,47]. *Domibacillus* was one of the most frequent aerobic genera detected, but little is known about the species of this genus [28]. Among the 20 most frequently detected anaerobic genera (Figure 3B), *Enterobacter*, *Aeromonas*, and *Bacillus* contain species that are both pathogenic and beneficial to the soil [26,27,28,34,35,36] whereas *Paeniclostridium* [50], *Hathewaya/Clostridium* [51], *Escherichia/Shigella* [43,44], and *Enterococcus* contain notable pathogens with no established roles in the soil [52]. Of the pathogenic genera, *Escherichia/Shigella* was the only genera that decreased in abundance in the exposed area. *Aeromonas*, *Bacillus*, and *Pseudomonas* are diverse genera that contain many beneficial soil bacteria in addition to potential human pathogens [26,27,40].

The decrease in the abundance of *Enterobacter*, *Aeromonas*, *Comamonas*, *Stenotrophomonas*, *Bordetella*, and *Staphylococcus* in areas exposed to pesticides may impair degradation of organic compounds, plant growth, microbial homeostasis, and plant protection from microbes and insects [26,31,33,36,41,53,54]. Conversely, *Domibacillus*, *Acinetobacter*, *Pseudomonas*, and *Bacillus* abundance in pesticide-exposed areas have the potential to promote bioremediation and biocontrol of the pesticide-contaminated field, improve mineralization, promote plant growth and nutrient mobilization [25,28,29,30,40].

Application of herbicides has been shown to induce stress conditions in non-photosynthetic microorganisms. For instance, metabolism of the Gram-negative bacteria *Stenotrophomonas maltophilia* usually present in rice field irrigation channels [42], (also identified in this study) has been demonstrated to be negatively affected by herbicides [55]. Also, a mixture of quinclorac and bensulfuron-methyl (BSM) (also applied in this study; Table 1) induced the activity of antioxidant enzymes superoxide dismutase and catalase of *S. maltophilia* strain WZ2 and thus, demonstrating the induced oxidative stress caused by the herbicides. The effect of BSM on soil microbial communities in a model paddy microcosm study showed that the nitrification potential was significantly suppressed [56]. In a related study, application of the recommended dose of bispyribac-sodium and a double dose altered soil bacterial populations, enzyme activities, and functional microbial diversity in a paddy soil [57]. Thus, we expect similar effects of the applied herbicides on the bacterial ecosystem of our study site.

In the natural environment, microorganisms have access to abundant and diverse array of carbon sources that may be easily assimilated than complex organic compounds. Biodegradation of 2,4-D is important in determining its overall fate in the environment, which is used on the rice field studied (Table 1). Degradation of 2,4-D in the soil is a fundamental attenuation process, which is influenced by both abiotic and biological processes. Different soil constituents and interactions of microbial communities and 2,4-D in soil play a critical role in the degradation process. 2,4-D usually degrades after a few days of its application through both abiotic and biotic interactions [58]. Soil microorganisms also play vital roles in the degradation of pesticides and mineralization of their metabolites. Among these microorganisms are dominant species of endophyte *Pseudomonas* (40%) and Enterobacter (18%) [59]. Pseudomonas is a diversified genus possessing a series of catabolic pathways and enzymes involved in pesticide degradation. Pseudomonas putida MAS-1 is reported to be efficient in chlorpyrifos degradation by a rate 90% higher than other species of Pseudomonas [60]. The chlorpyrifos degradation involve the metabolism and mineralization of 3, 5, 6-trichloro-2-pyridinol and 3,5,6-trichloro-2-methoxypyridine. Pseudomonas is the group of bacteria present in large amount in the soil and have a vital role in the mineralization of organic matter. They are metabolically adaptable and have capability to degrade most of the aromatic hydrocarbons, oil, petroleum products, and pesticides [61]. Pseudomonas has the capability to mineralize phenolic compounds [62]. A variety of low-molecular-weight compounds, including chlorinated aliphatic hydrocarbons can also be metabolized by Pseudomonas because of diversified range of catabolic pathways. Our study however did not support the ability of Pseudomonas to degrade pesticides, as there was no significant difference in the abundance of Pseudomonas genus between the exposed and unexposed areas (Figure 3A).

In order to reduce the effect of pesticides on bacterial diversity, it is important to monitor the response of soil bacterial communities and various enzymatic activities. Various bacterial genera were negatively impacted in the area exposed to pesticides and most of these genera are known to be involved in nutrient mobilization, plant growth promotion, mineralization, and metabolism of organic compounds. It remains to be seen whether the depletion of soil microbes would also affect the fertility of the soil and overall productivity of this rice field. The increase in abundance of the genera *Domibacillus*, *Bacillus*, and *Clostridia* suggest they were generally not constrained by the pesticides. It is possible that these bacteria can metabolize the pesticides or require a much higher concentration of the pesticides in order to be affected. Ongoing research aims to identify the species among these genera present in the sample and to explore their potential as candidates for bioremediation. Our study shows that there is a need to educate and encourage farmers to adopt innovative integrated pest management strategies that promote the function of beneficial microbes with little to no deleterious impact on the soil bacterial ecosystem.

## Figures and Tables

**Table 1:** The list of pesticides, application rate per hectare, active ingredients, and formulations used on the irrigation field under study.

Pesticide Name	Active Ingredient Conc.	Application Rate/ha	Target Pest/Disease	Application Method	Frequency
Condex	Bensylfuron methyl (30%)	0.42 Kg	Selective herbicide	spraying	3
Kilsect 2.5 EC	Lambda Cyhalothrin (25 g/L)	1.0 L	Grasshoppers, Worms, Thrips	spraying	5
Bounty/Nakitse	Bispyribac sodium (400 g/L)	62.5-75 Ml	Selective herbicide	spraying	3
Nativo	Tebuconazole (200 g/L)	1.0 L	Blast	spraying	5
	Trifloxystrobin (100 g/L)				
Orizo plus	Propanil (360 g/L)	2.0 L	Selective herbicide	spraying	3
	2,4,D amine (200 g/L)				
Dursban/Sunpyrifos	Chlorpyrifos (480 g/L)	1.0 L	Grasshoppers, Worms, Thrips	spraying	5
Allogator	Pendimethalin (400 g/L)	3.0 L	Selective herbicide	spraying	3

**Table 2:** Description of the most prevalent bacteria genera detected in the soil samples.

Genus	Respiration	Habitat	Role in Soil	Potential Pathogen	Open Literature References	* Zone of Highest OTU
*Acinetobacter*	Obligate aerobes	Soil, water	Mineralization	Pathogenic	[25]	Residual
*Aeromonas*	Facultative anaerobes	Soil, water	Microbial equilibrium	Pathogenic	[26]	Unexposed
*Bacillus*	Obligate aerobes Facultative anaerobes	Ubiquitous	Plant protection from plants and insects	Pathogenic	[27,28,29,30]	Residual
*Bordetella*	Aerobes	Soil, water, sediment, plants	Possible degradation of organic compounds	Pathogenic	[31]	Unexposed
*Chitinophagaceae* (Unc40442)	Facultative anaerobes	Soil	Chitin degradation	Not Pathogenic	[32]	Exposed
*Comamonas*	Facultative anaerobes, Aerobes	Soil, water	Possible degradation of organic compounds	Pathogenic	[33]	Unexposed
*Enterobacter*	Facultative anaerobes	Ubiquitous	Plant growth regulator	Pathogenic	[34,35,36]	Unexposed
*Leucobacter*	Aerobes	Soil, sediment, water	Possible bioremediation	Pathogenic	[37]	Unexposed
*Paenibacillaceae* (GIWBac55)	Facultative anaerobes	Soil, water, plants	Nitrogen fixation, plant growth, plant protection from microbes and insects	Pathogenic	[38]	Unexposed
*Paenibacillus*	Facultative anaerobes	Soil, water, plants	Nitrogen fixation, plant growth, plant protection from microbes and insects	Pathogenic	[38]	Unexposed
*Pseudarthrobacter*	Obligate aerobes	Soil	Possible biodegradation of organic compounds	Pathogenic	[39]	Residual
*Pseudomonas*	Facultative anaerobes, aerobes	Ubiquitous, Soil, water, plants, rhizosphere	Biocontrol, Plant Growth promotion, nutrient mobilization, soil bioremediation	Pathogenic	[40]	Residual
*Staphylococcus*	Facultative anaerobes	Ubiquitous, Soil, water	Possible degradation of organic compounds, Plant Growth promotion	Pathogenic	[41]	Unexposed
*Stenotrophomonas*	Aerobes	Soil	Plant protection from plants and insects	Pathogenic	[33,42]	Unexposed
*Escherichia/Shigella*	Aerobes	Ubiquitous	Plant growth promoter	Pathogenic	[43,44]	Exposed
*Domibacillus*	Aerobes	Ubiquitous, Soil, water	Unknown	Unknown	[28]	Residual
*Halalkalibacillus*	Aerobes	Soil, water	Unknown	Pathogenic	[45]	Exposed
*Vogesella*	Aerobes	Soil, water sediments	Unknown	Not Pathogenic	[46,47]	Exposed
*Pasteurella*	Facultative Aerobes	Soil, water	Biocontrol	Pathogenic	[48]	Exposed
*Bergeyella*	Aerobes	Soil, water	Unknown	Pathogenic	[49]	Exposed

* Zone of highest OTU of the study area.
